# SLC20A1 is a prospective prognostic and therapy response predictive biomarker in head and neck squamous cell carcinoma

**DOI:** 10.18632/aging.205597

**Published:** 2024-02-26

**Authors:** Xiajing Qian, Ming Jin, Yanping Bei, Chongchang Zhou, Shuai Fang, Kaitai Liu

**Affiliations:** 1 Department of Radiation Oncology, The Affiliated Lihuili Hospital, Ningbo University, Ningbo, Zhejiang, China; 2 Department of Otorhinolaryngology Head and Neck Surgery, The Affiliated Lihuili Hospital, Ningbo University, Ningbo, Zhejiang, China; 3 Department of Thoracic Surgery, The Affiliated Hospital of Medical School of Ningbo University, Ningbo, Zhejiang, China

**Keywords:** SLC20A1, squamous cell carcinoma, prognosis, TCGA, cancer

## Abstract

Background: SLC20A1, a prominent biomarker in several cancers, has been understudied in its predictive role in head and neck squamous cell carcinoma (HNSCC).

Methods: The Cancer Genome Atlas (TCGA) database was used to analyze HNSCC prognosis, SLC20A1 overexpression, and clinical characteristics. Quantitative real-time PCR and Western blot analysis confirmed SLC20A1 expression in HNSCC tissues. Cellular behaviors such as invasion, migration and proliferation were assessed using Transwell, wound healing and colony formation assays. Immune system data were obtained from the Tumor Immune Estimation Resource (TIMER) and CIBERSORT databases. Gene Ontology (GO), Kyoto Encyclopedia of Genes and Genomes (KEGG), and Gene Set Enrichment Analysis (GSEA) were used to explore biological parameters and pathways associated with SLC20A1 overexpression in HNSCC.

Results: In 499 HNSCC samples, SLC20A1 mRNA and protein expression were significantly higher than in 44 normal counterparts, confirmed by 24 paired samples. Patients were categorized based on SLC20A1 levels, survival status and overall survival. High SLC20A1 expression correlated with advanced T stage, increased risk scores and decreased survival. Stage, age and SLC20A1 expression emerged as independent predictive factors for HNSCC in univariate and multivariate analyses. SLC20A1 overexpression, which is associated with poor prognosis, may influence cell proliferation, migration, invasion, chemotherapy response, and the immune milieu.

Conclusions: SLC20A1 overexpression in HNSCC, characterized by increased cellular invasion, migration and proliferation, is a potential prognostic biomarker and therapeutic response indicator.

## INTRODUCTION

Worldwide, head and neck squamous cell carcinoma (HNSCC) is the 6^th^ commonest malignancy which had been paid more attention to, including oral and maxillofacial cancer, laryngeal cancer, salivary gland cancer, oropharyngeal cancer and hypopharynx cancer [1]. HNSCC incidence is continuously rising and is estimated to rise by 30% until 2030 [2]. Furthermore, the5-year survival rate was estimated for HNSCC cases has been estimated to be < 50%, despite improved multimodality treatments in past decades [3]. The lack of rapidly improving patient survival and personalized treatment approaches has propelled research into the molecular landscape of HNSCC. Identification of potential prognostic markers associated with treatment benefit can allow individualization of therapy for patients with HNSCC. Thus, effective prognostic and therapeutic indicators are urgently needed.

It is well-known that SLC20A1 and SLC20A2 form the SLC20 family, and SLC20A1 has been considered as a secondary-active, Na^+^-dependent cotransporters to transport inorganic phosphate (Pi) through the cell membrane, which favorably comprises two sodium ions, and monovalent inorganic phosphate (H_2_PO4^−^) [4]. Pi acts as a basic nucleotides and phospholipids component, and participates in several cell functions, such as energy metabolism and cellular signaling pathways [5, 6]. Studies reported that tumor tissues contain high Pi traces, making them potential indicators of tumor prognosis that might independently promote the cell proliferation [7]. SLC20A1, which was commonly known as PiT-1, is involved in a wide variety of cellular processes, like cell development, ability to differentiate and proliferate, adhesion, and apoptosis [7–12]. Due to a lack of pro-B cells, mice missing SLC20A1 were found to be highly lymphopenic, and a slight neutropenia. This trait, which is exclusive to the hematopoietic pathway, is linked to a malfunction in cellular division [12]. Some results also give important insights into the pathophysiology and therapy of myelosuppression by showing that Pi metabolism and hematopoietic stem cell survival are frequently related through the Akt/p53-SLC20A1 axis [13]. In recent years, investigations showed the vital role that SLC20A1 plays in the progression of various tumors, such as pituitary tumors, breast cancer, and tongue SCC, indicating the high expression of SLC20A1 in many tumors and its potential association with unfavorable prognosis [14–17]. More advanced stages of tumor luminal A breast cancer were associated with increased SLC20A1 expression, according to a previous investigation. Moreover, this SLC20A1 high subset of individuals showed worse responses to endocrine treatment, particularly in breast cancer luminal A and B subtypes [18]. Nonetheless, the association between SLC20A1 and the prognosis of patients with HNSCC are rarely reported.

As systematic interpretations of SLC20A1 in HNSCC being undefined, we utilized bioinformatics analysis tools and laboratory experiments to explore the expression and multilevel clinical value of SLC20A1 in HNSCC. This study projects to investigate the impact of SLC20A1 in tumor progression, prognostic value, molecular mechanism and treatment response in HNSCC.

## MATERIALS AND METHODS

### Data collection

The Cancer Genome Atlas (TCGA) database was used as the source from which data on SLC20A1 expression, prognostic information and clinical features of HNSCC cases was extracted (https://xena.ucsc.edu/). Normalized gene transcript data from Fragments Per Kilobase of transcript per Million mapped reads (FPKM) were involved in this study. The clinicopathological features of the included specimens, like gender, age, grade, node (N) and tumor (T) statuses, and stage were used in the analyses. Another 24 paired tissues were stored in our laboratory. The Ethics Committee of the Affiliated Lihuili Hospital has granted authorization for the conduct of this research, under the approval number 2022SL415-01.

### Expression difference of the SLC20A1 and its association with DNA methylation in HNSCC

Perl software was used to extract the SLC20A1 mRNA expression level in HNSCC from the HTSeq data. R software (version 3.6.0; *limma* package) is applied for analysis of the differential expression of the SLC20A1 in HNSCC samples by comparing it to normal tissues. In addition, the methylation of these cg sites (cg05422897, cg05672265, cg06121808, cg10762132, cg10898730, cg23886783, cg26703507) in SLC20A1 gene’s promoter regions was obtained by downloading DNA methylation data from Illumina's Human Methylation 450K data. Annotation data used for cg sites were downloaded from Illumina's help desk (https://support.illumina.com). Utilizing the Pearson correlation with the R package *corrplot*, our team analyzed the correlation between DNA methylation and SLC20A1 mRNA expression.

### Prognosis value of SLC20A1 in HNSCC

Using the optimal threshold for SLC20A1 expression identified by the iterative algorithm of the *survival* package, we constructed the overall survival (OS) curves using the Kaplan-Meier method for a more precise analysis. A risk assessment model for prognosis prediction was generated to bind the expression level with survival outcome of each patient. TCGA datasets were used to perform all analyses. The independent prognostic value of SLC20A1 in HNSCC was assessed using uni- and multivariate Cox regression analyses. Two R packages, *survminer* and *survival,* were used for all these analyses and the *ggplot* was used for the results represented by forest plot.

### Nomogram construction for prognosis prediction

A nomogram was constructed to provide a quantitative approach for predicting survival probability in HNSCC, which comprised the SLC20A1 clinical, pathological, and expression factors, including gender, age, grade, stage, T status and N status, by using R package *rms*. In the nomogram, we used the OS rates at 1, 3, and 5 years as endpoints. Area under the curve (AUC) and receiver operating characteristic (ROC) curve were developed to evaluate these rates’ predictive ability. Furthermore, calibration plots were drawn to assess the nomogram accuracy for internal validation [19].

### Prediction of therapeutic sensitivity in HNSCC with different expression of SLC20A1

To evaluate the chemoresistance in HNSCC, the 50% inhibiting concentration (IC50) value of the 3 medicine which was mainly used in chemotherapy for HNSCC was inferred using the pRRophetic algorithm. The TIDE score uses gene expression patterns to predict clinical outcome for immune checkpoint inhibitors (ICIs) [20]. A lower TIDE score is associated with a greater immunotherapy response.

### Expression of SLC20A1 and the association with tumor infiltrating immune cells (TIICs)

The relationship between SLC20A1 and 22 subsets of tumor-infiltrating lymphocytes (mostly neutrophils, macrophages, dendritic cells, CD8+ and CD4+ T cells, and B cells) were evaluated using the CIBERSORT database (https://cibersort.stanford.edu/). For this purpose, we utilized the Tumor Immune Estimation Resource (TIMER) database (https://cistrome.shinyapps.io/timer/) to investigate TIICs and SLC20A1 expression in depth.

### Functional enrichment analysis

Enrichment studies of the thematic expression of SLC20A1 were performed to identify the possible molecular processes and roles of SLC20A1 in HNSCC. Two further studies, using the Kyoto Encyclopedia of Genes and Genomes (KEGG) and the Gene Ontology (GO), were conducted. The R programs cluster profiler and enrichplot were applied to run KEGG pathway and GO functional enrichment studies. To investigate potential signaling pathways in which SLC20A1 participates, a gene set enrichment analysis (GSEA) was run. Remarkably variable expression levels were defined as having a false discovery rate (FDR) of < 0.05 and a | log2FC | ≥ 1.

### Cell culture and transfection

TU686 (laryngeal squamous cell carcinoma cell line) and CAL-27 (tongue squamous cell carcinoma cell line) human HNSCC cells were employed in this investigation. HNSCC cells were cultured in 0.05 mg/mL gentamycin (Schering-Plough Europe, Belgium) and RPMI 1640 with 10% FBS. Saturated humidity with 5% CO_2_, and 37° C was used to cultivate cells. GeneChem created SLC20A1-specific siRNAs (si-SLC20A1#1/2/3) and si-NC (China). These plasmids were introduced into CAL-27 and TU686 cell lines using Lipotransfectamine 3000 (Thermo Fisher Scientific, USA).

### CCK-8 experiment and colony formation assay

Cell lines CAL-27 and TU686 were grown in 96-well plates at a density of 5×10^3^ cells/well for 0, 24, 48, 72, and 96 hours. After two hours in the dark at 37° C, cells were counted using a cell counting kit 8 (CCK-8) (Dojindo, Japan). Cell absorbance was measured at a wavelength of 450 nm. Six-well plates were seeded with CAL-27 and TU686 cells (at a density of 500 cells per well), and the media were replaced every four days. Colonies were fixed in 4% paraformaldehyde for 30 minutes and dyed with 0.1% crystal violet (Sigma-Aldrich, USA) for 15 minutes after being incubated for two weeks. The colony count was performed using Image J program.

### Wound healing assay

A six-well plate was used for the cellular seeding. After the cells had adhered together to create a monolayer, a scratch was made in it using the tip of a plastic pipette with 200 L. Cells should be washed with PBS three times. After discarding the whole DMEM, 2 mL of DMEM devoid of serum was applied to each well. Using an IX71 inverted microscope (Olympus, Japan), we photographed the drawn distance of each hole at the stated periods (0 h, 48 h) and analyzed the photos using Image J. There were three rounds of each test.

### Transwell assay

By a 24-well Transwell chamber, a Transwell experiment was done to test how well the cells could invade. TU686 and CAL-27 cell lines in media without serum were put in top chambers that had been covered with 2% Matrigel (BD Biosciences, USA). In the bottom chambers, medium with 20% FBS was added. After two days of incubation, 4% paraformaldehyde was used to fix the cells that had moved to the bottom of the membrane. The cells were fixed, and then crystal violet was used to stain them for 15 minutes. With the use of Image-Pro Insight software and an Olympus IX71 microscope from Japan, the findings were seen and recorded (Olympus, Japan).

### Quantitative real-time polymerase chain reaction (qRT-PCR)

Using TRIZOL (Invitrogen, USA), total RNA was extracted from 24 HNSCC and normal samples, and then cDNA was synthesized using a reverse transcription kit (Novoprotein, China). The qRT-PCR reactions were performed using a LightCycler 480 (Roche, USA) instrument and NovoStart SYBR qPCR (Novoprotein, China), as per the manufacturers' instructions. During each of the 35 cycles, the reaction was subjected to the following conditions: heating, denaturation, annealing, and extension at 95° C, 60° C, 72° C, and 95° C for 30 s, 10 s, 30 s, and 30 s, respectively. The SLC20A1 relative expression was determined using the two-fold comparative Ct technique (2Ct). Each sample has three independent tests conducted on it. These were the primer sequences that were provided: SLC20A1, F: 5’-TGGCAACGCTGATTACCAGT-3’; R: 5’-CAGCCCTTGAGTCGAGTTGT-3’. GAPDH, F: 5’-TCAAGATCATCAGCAATGCC-3’; R: 5’-CGATACCAAAGTTGTCATGGA-3’.

### Western blotting analysis

Tissue was collected, lysed, and protein content was determined using a BCA protein assay kit (Beyotime, China) per the manufacturer's instructions. Using a 10% sodium dodecyl sulfatepolyacrylamide gel, we separated the protein that had been extracted using the improved RIPA buffer (Beyotime, China). The membrane was then transferred onto a PVDF membrane (Millipore, USA) at a continuous current of 240 mA for 2 hours, followed by blocking in 5% skim milk for 2 hours and incubation with anti-SLC20A1 primary antibody (Proteintech, China) at 4 degrees Celsius for 1 night. Following a 3-time TBST wash, the membrane was incubated with a secondary antibody from Proteintech (China) for an hour. Eventually, chemiluminescence (Advansta, USA) was used to identify and visualize the target protein with Image Lab software.

### Statistical analysis

Documents for GESA preparation, DNA methylation, and HTSeq FPKM were parsed using Perl software version 5.32 to glean the relevant data. Differential expression, nomogram generation, prognostic value assessment, and Pearson correlation were all performed using R 4.0.3 program with specialized packages. Appropriate testing methods were selected for comparison based on the type of samples, including the T-test, Chi-square test, and Wilcoxon test. A significance level of 0.05 was used.

### Data availability

The data that supported the findings of this study are openly available from The Cancer Genome Atlas (TCGA) program (https://portal.gdc.cancer.gov/).

## RESULTS

### HNSCC prognosis and SLC20A1 expression

**Figure 1:**
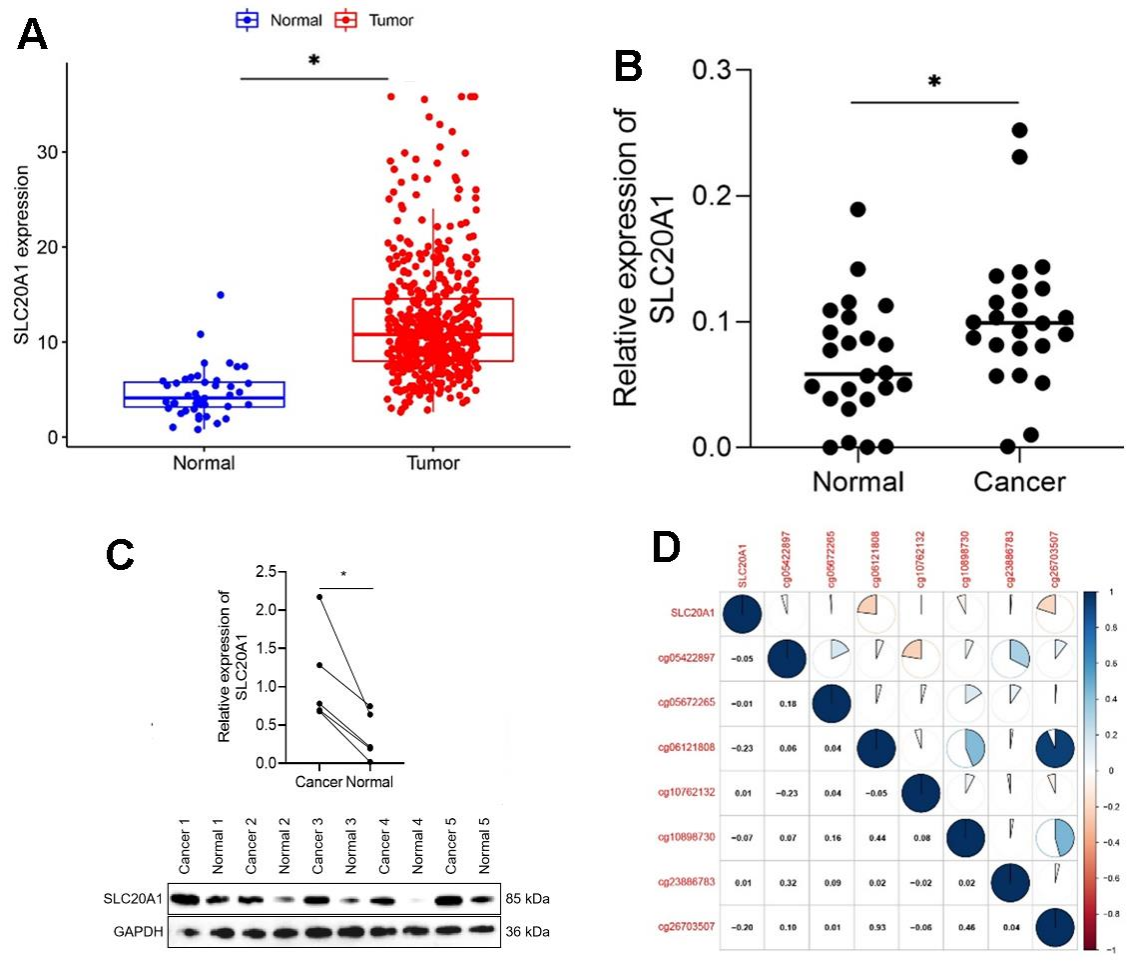
**Exaggerated SLC20A1 expression levels in HNSCC.** (**A**, **B**) SLC20A1 mRNA expression between the HNSCC specimens and nearby sound tissues from TCGA database and qRT-PCR analysis. (**C**) SLC20A1 protein expression in the HNSCC specimens and nearby sound ones from Western blotting analysis. (**D**) DNA methylation modification associated with SLC20A1 expression in HNSCC. *, *P*<0.05.

We analyzed 383 cases to see whether or not there was a correlation between SLC20A1 expression and patient features. It was found that patients that expressed a lot of SLC20A1 had a higher-than-average T stage, as shown in Table 1 (P < 0.05). Our findings demonstrated the significantly higher expression levels of SLC20A1 mRNA in 499 HNSCC samples compared to 44 adjacent normal ones, obtained from the TCGA database (Figure 1A, *P*<0.05). qRT-PCR analyses of 24 pair-matched samples stored in our laboratory consistently showed a remarkable increase of SLC20A1 mRNA in HNSCC tissues (Figure 1B, *P*<0.05). Western blot of five paired tissues showed that SLC20A1 protein was highly expressed in HNSCC tissues (Figure 1C). Furthermore, SLC20A1 methylation status in promoter cg sites was also investigated, as was the correlation with gene expression. From Pearson correlation, we could obtain that the abnormal DNA methylation might lead to the high expression of SLC20A1 (Figure 1D).

**Table 1:** SLC20A1 overexpression association with the clinical and pathological features of the included HNSCC cases.

**Variables**	**Cases (n)**	**SLC20A1**		***P-value***
		**High**	**Low**	
Total	383	169	214	
*Age (years)*				
>65	129	55	74	0.676
≤65	254	114	140	
*Gender*				
Male	281	125	156	0.814
Female	102	44	58	
*Pathological stage*				
I/II	69	28	41	0.512
III/IV	314	141	173	
*T stage*				
T1/T2	134	48	86	0.016*
T3/T4	249	121	128	
*N status*				
Negative	162	72	90	0.914
Positive	221	97	124	
*Histologic grade*				
G1-2	293	124	169	0.199
G3-4	90	45	45	

*, *P*<0.05.

**Figure 2:**
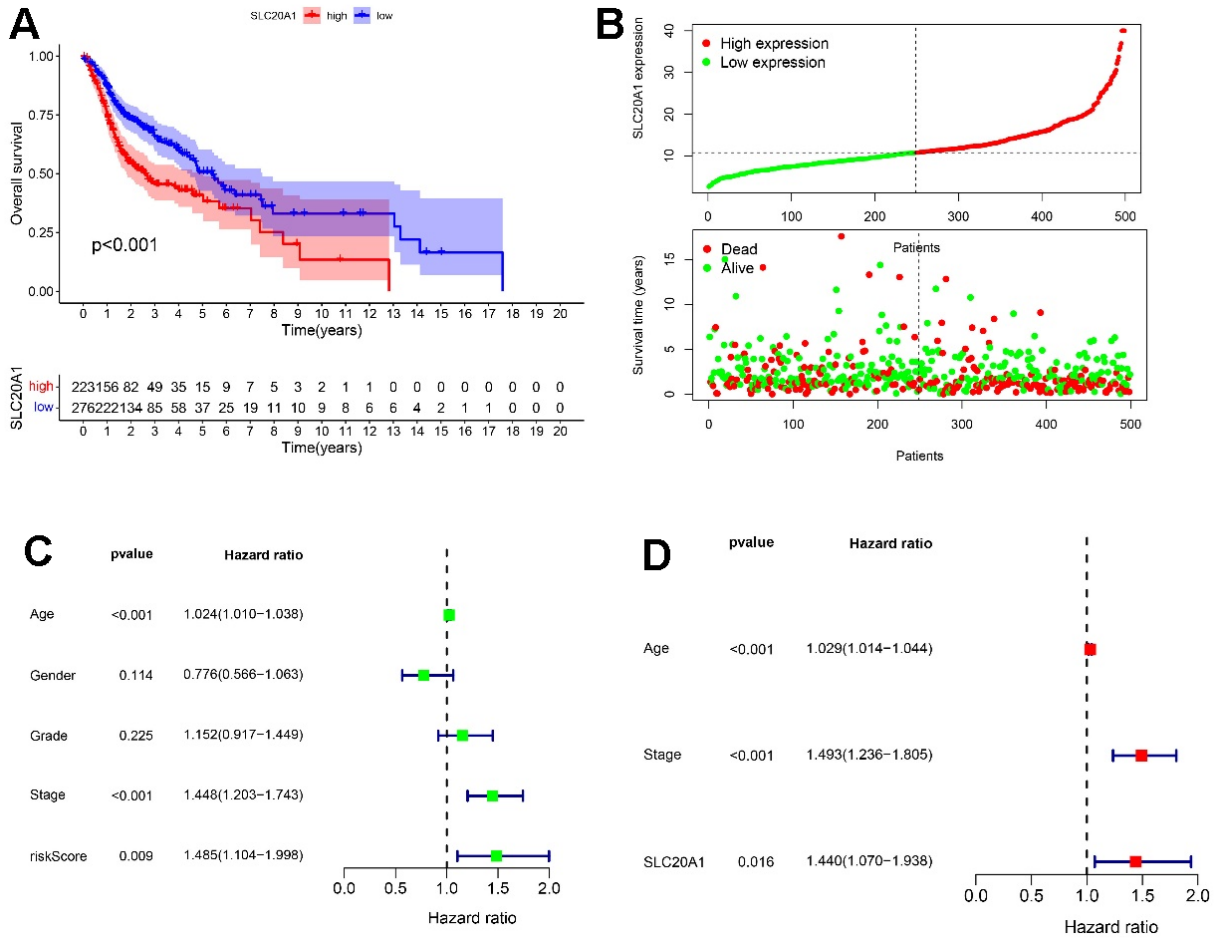
**HNSCC prognosis via SLC20A1.** (**A**) Kaplan-Meier analysis of poor survival outcomes with SLC20A1 overexpression in HNSCC from TCGA database. (**B**) Risk score with survival time in TCGA database. (**C**, **D**) Forest plots representing the uni- and multivariate analysis of the significantly marked prognostics.

Additional investigation of SLC20A1's relevance to survival in HNSCC was performed using Kaplan-Meier analysis. We determined the threshold for separating patients into those with high or low SLC20A1 expression based on SLC20A1 expression level, survival status, and OS time. The findings showed that individuals having a high SLC20A1 expression had a higher risk score, and a shorter OS time (Figure 2A, 2B). In addition, stage, age, and SLC20A1 expression were shown to be independent predictive variables for HNSCC in both uni- and multivariate analyses using Cox regression (Figure 2C, 2D).

### Silencing SLC20A1 inhibited cell invasion, migration, and proliferation in HNSCC

**Figure 3:**
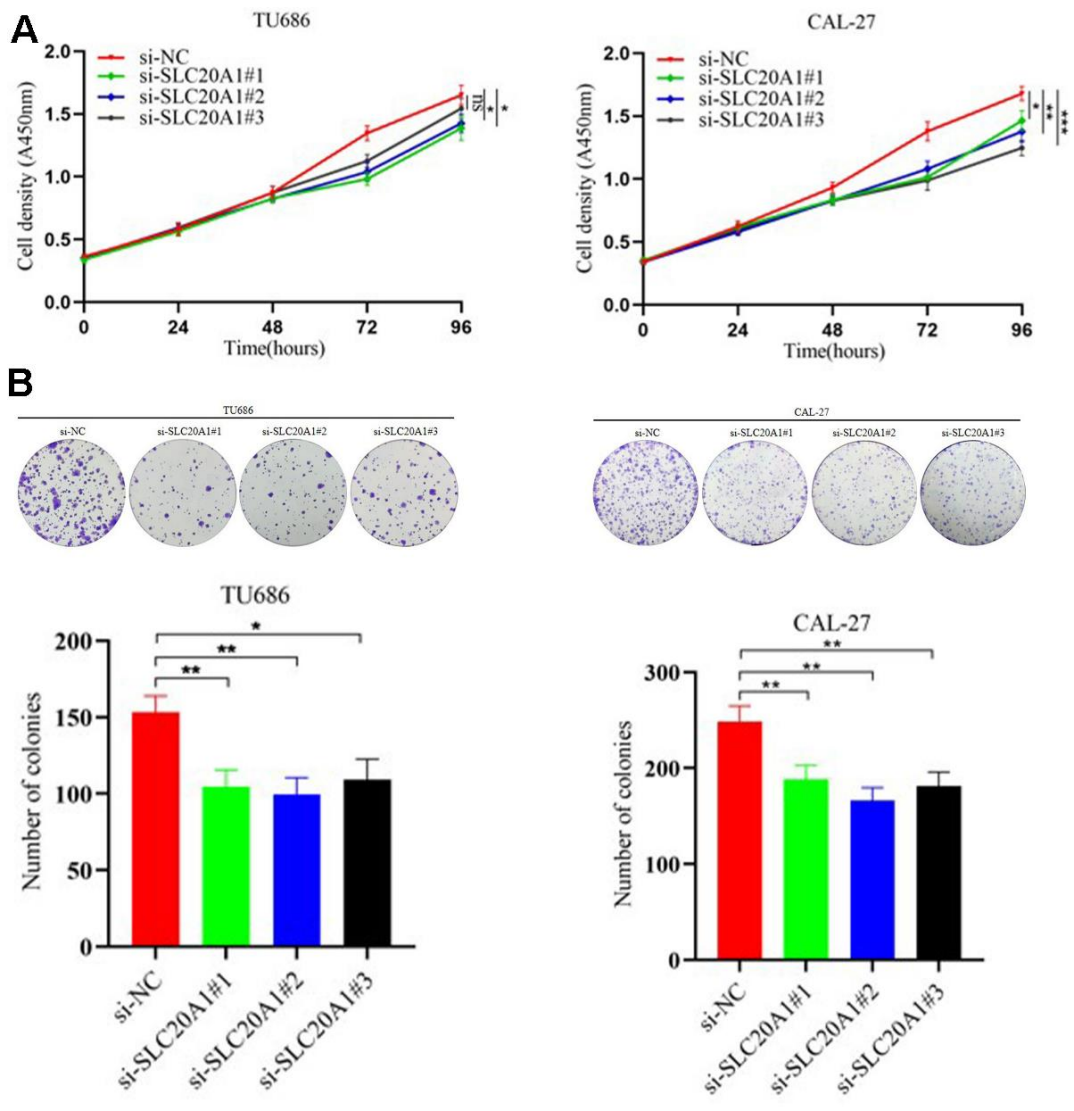
**Silencing SLC20A1 inhibited cell proliferation in TU686 and CAL-27 cells lines.** (**A**) CCK8 and (**B**) colony formation experiments showed that silencing SLC20A1 inhibited cell proliferation. *, *P*<0.05. **, *P*<0.01. ***, *P*<0.001.

**Figure 4:**
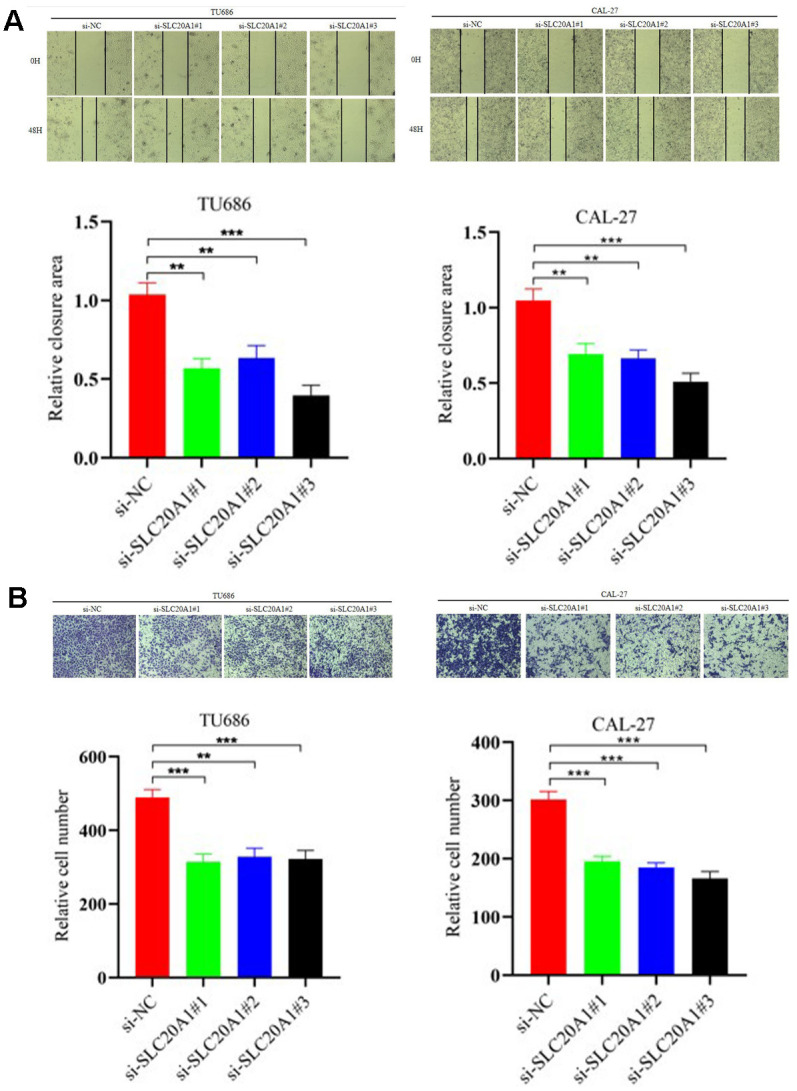
**Silencing SLC20A1 inhibited migration and invasion in HNSCC.** (**A**) Wound-healing experiments showed that silencing SLC20A1 inhibited cell migration. (**B**) Silencing SLC20A1 inhibited the ability to invade CAL-27 and TU686 cell lines. *, *P*<0.05. **, *P*<0.01. ***, *P*<0.001.

In our research, we used the CAL-27 and TU686 cell lines to examine SLC20A1's role in HNSCC cells. Transfection of siRNA for nucleotides cofactors (NC) and SLC20A1 were performed in CAL-27 and TU686 cell lines, respectively. Cell proliferation was reduced in both cell lines treated with si-SLC20A1 compared to controls, as measured by cell counting tests and colony formation (Figure 3, P<0.05). The wound healing experiments were utilized to measure the migratory potential of HNSCC cells, and the findings indicated a substantially smaller wound area in the controls compared to cases in the si-SLC20A1 groups (Figure 4A, P<0.05). Furthermore, Transwell assay findings showed that SLC20A1-down-expressed groups were less invasive than controls (Figure 4B, P<0.05). Finally, we hypothesized that knocking down SLC20A1 in HNSCC cells would reduce their ability to proliferate, migrate, and invade.

### Nomogram development

**Figure 5:**
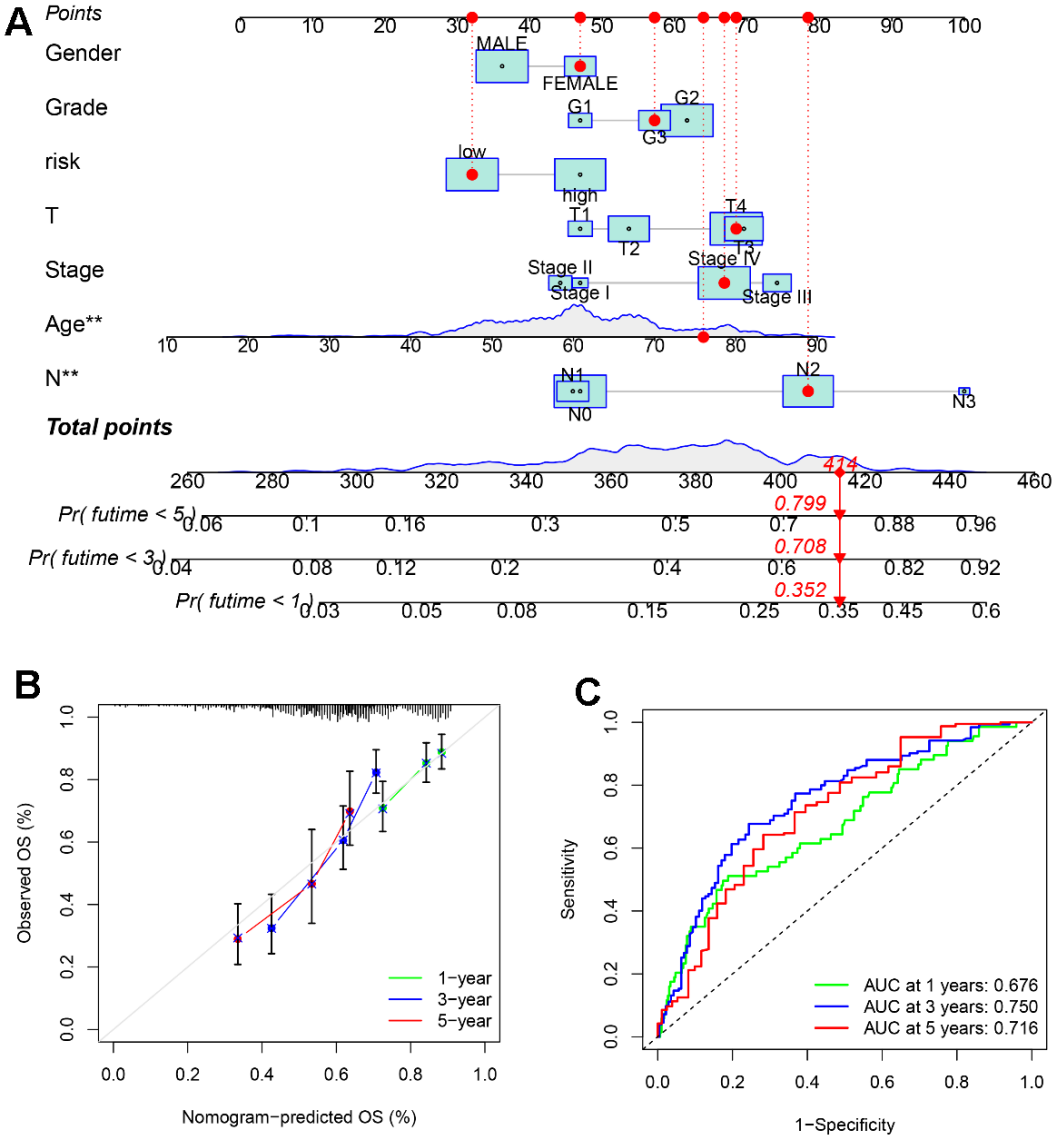
**A nomogram based on SLC20A1 expression and clinical features.** (**A**) Nomogram to predict the overall survival (OS) of the 1^st^, 3^rd^, and 5^th^ years for HNSCC cases. (**B**) Calibration curve of the nomogram prognostic model. (**C**) ROC curve of the nomogram for predicting the overall survival (OS) of the 1^st^, 3^rd^, and 5^th^ years.

A nomogram has been established based on SLC20A1 expression and clinical characteristics (Figure 5A), as a quantitative approach for HNSCC prognosis prediction. Calibration curve indicates good agreements between the observed outcome and predicted probability (Figure 5B). For further validation of the prognostic power of combined clinical features and SLC20A1 expression, ROC curve has been created and the AUC for survival rates at 1, 3, and 5 years were 0.676, 0.750 and 0.716, with moderate prediction accuracy (Figure 5C), indicating the significant ability of the developed monogram for predicting HNSCC prognosis.

### The association between SLC20A1 overexpression with chemotherapy and immunotherapy response in patients with HNSCC

**Figure 6:**
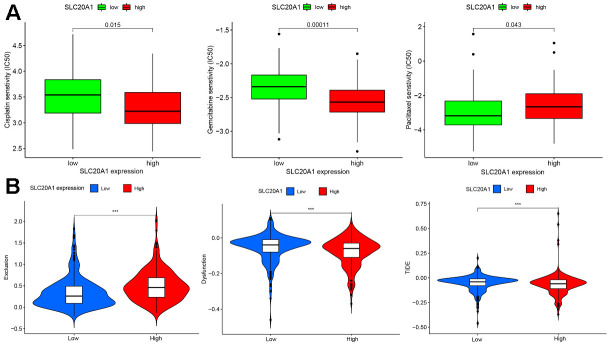
**Chemotherapy and immunotherapy response were associated with SLC20A1 expression in HNSCC.** (**A**) Chemotherapeutic responses with differential SLC20A1 expression in HNSCC. (**B**) Correlation between SLC20A1 expression and immunotherapy responses. *, *P*<0.05. **, *P*<0.01. ***, *P*<0.001. IC50, 50% inhibiting concentration.

As shown in Figure 6A, SLC20A1 expression was remarkably associated with cisplatin, gemcitabine, and paclitaxel. Moreover, SLC20A1 overexpression was more sensitive to cisplatin and gemcitabine, while low expression of SLC20A1 were more sensitive to paclitaxel.

**Figure 7:**
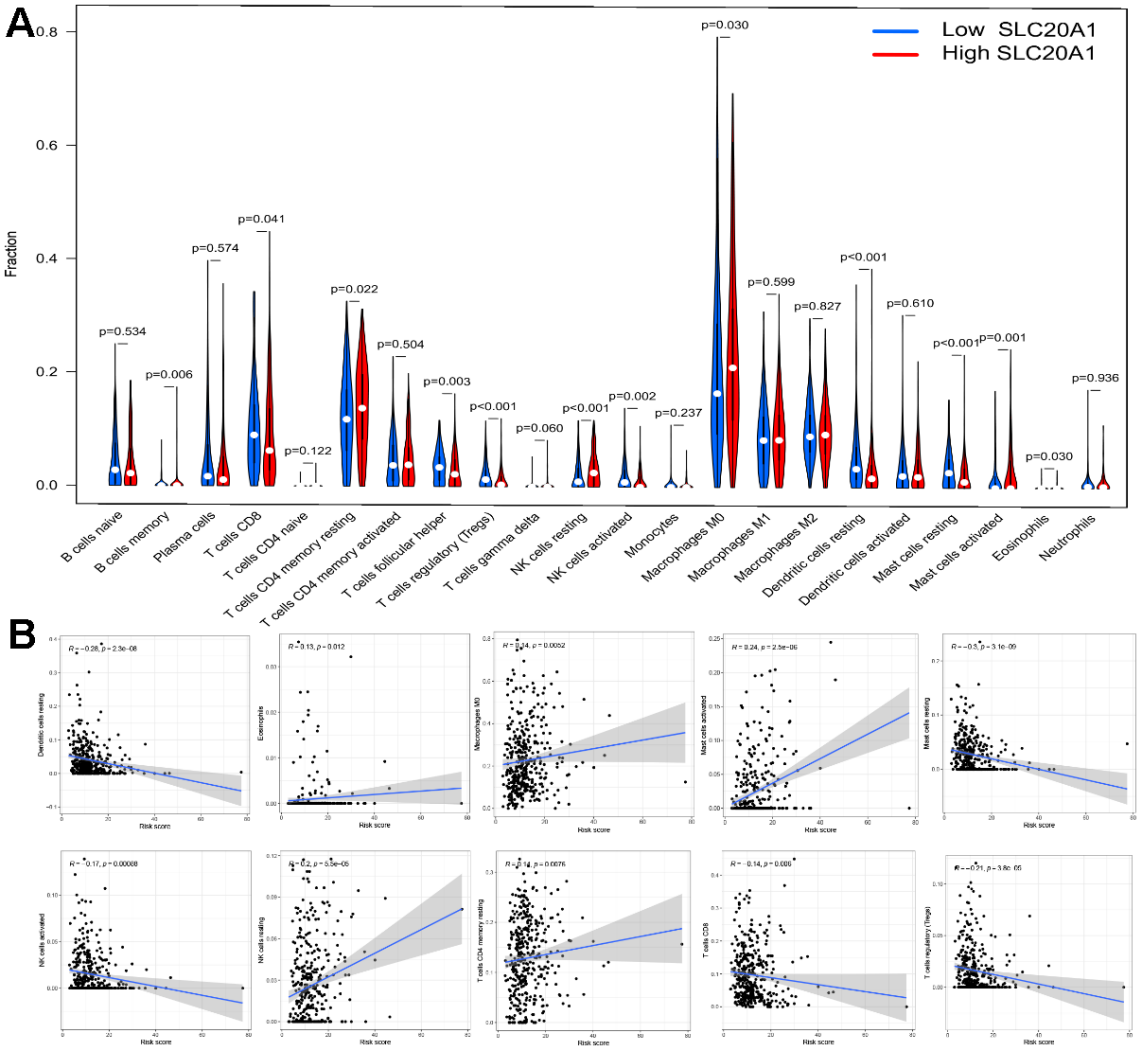
**Association between SLC20A1 expression and TIICs in HNSCC.** (**A**) Abundance of TIICs infiltration between low and high SLC20A1 expression in HNSCC from CIBERSORT database. (**B**) Correlation between SLC20A1 expression and TIICs from TIMER database.

The results demonstrated that SLC20A1 overexpression was associated with more immune exclusion and dysfunction, and a lower TIDE score, indicating that patients with a higher SLC20A1 expression level are less sensitive to immunotherapy (Figure 6B). Specifically, we looked at how SLC20A1 expression relates to TIICs in HNSCC. SLC20A1 expression variation was strongly correlated with a wide range of immune cell types (Supplementary Figure 1). In our analysis of the TIMER and the CIBERSORT databases, we found that high SLC20A1 expression was negatively correlated with resting mast cells, dendritic cells, resting NK cells, T cells regulatory (Tregs), and resting CD8+ T cells, and positively correlated with Eosinophils, activated mast cells, macrophages M0, resting NK cells, and CD4+ memory T cells (Figure 7A, 7B). This led us to hypothesize that SLC20A1 expression has a role in modulating the immunological microenvironment in HNSCC, which is important for further studies.

### SLC20A1-related underlying molecular mechanism in HNSCC

A protein-protein interaction (PPI) network was constructed using the GENEMANIA database, followed by a Pearson correlation analysis based on gene expression data from TCGA. This analysis revealed a strong correlation between SLC20A1 and SLC20A2, especially in shared protein structural domains (Supplementary Figure 2). Analyses of GO functions and KEGG pathways were used to uncover a possible molecular mechanism involving SLC20A1 in HNSCC. The findings of the GO analysis revealed the correlation between cellular component, biological process and molecular function, and SLC20A1 expression (Supplementary Figure 3A, 3B). Meanwhile, the results from KEGG enrichment analysis revealed a remarkably enrichment of SLC20A1 in immune process and several pathways, including “IL−17 signaling pathway”, “cytokine−cytokine receptor interaction”, “TNF signaling pathway”, “NOD-like receptor signaling pathway”, “NF-κB signaling pathway”, “JAK-STAT signaling pathway”, “Chemokine signaling pathway” (Supplementary Figure 3C, 3D). According to the initial screening of pathways, the TNF signaling pathway has garnered our attention. Our bioinformatics analysis revealed significant overexpression of TNF and TNFR2 in head and neck squamous cell carcinoma (Supplementary Figure 4). Furthermore, there was a notable positive correlation between the expression of SLC20A1 and both TNF and TNFR2. *In vitro* experiments with the head and neck squamous carcinoma cell line TU686 showed that knocking out SLC20A1 significantly reduced TNF and TNFR2 expression levels compared to the control group (Supplementary Figure 5). Hence, we hypothesize that SLC20A1 may be involved in the regulation of the TNF/TNFR2 signaling pathway.

In addition, GSEA interpretation indicated that SLC20A1 overexpression had markedly positive correlations with numerous enrichment pathways (Supplementary Figure 6A, 6B), including “bladder cancer”, “colorectal cancer”, “renal cell cancer”, “small cell lung cancer”, “cell cycle”, “natural killer cell mediated cytotoxicity”, “P53 signaling pathway” and “MAKP signaling pathway”, In contrast, SLC20A1 low expression was significantly related with several metabolism and biochemical processes, such as “alpha linolenic acid metabolism”, “drug metabolism cytochrome P450”, “arachidonic acid metabolism” and “oxidative phosphorylation” (Supplementary Figure 6C). Accordingly, SLC20A1 might act as a novel biomarker in tumor progression and immune environment in HNSCC.

## DISCUSSION

In our study, results showed that SLC20A1 was remarkably upregulated in HNSCC tissues than adjacent normal ones at the mRNA level in the TCGA database. qRT-PCR analyses also confirmed the results. Protein level clear overexpression was also exhibited by Western blotting. According to Pearson correlation, DNA methylation was suggested as a reason for this upregulation. Subsequently, the clinical prognostic value of SLC20A1 was explored. According to Kaplan-Meier analysis, OS was significantly short among cases with SLC20A1 overexpression. Moreover, uni- and multivariate analysis indicated the prognostic power of SLC20A1 for HNSCC. Moreover, the results showed that overexpression of SLC20A1 was prominently related to high T stage. A significant diagnostic value for HNSCC was also demonstrated for SLC20A1 on ROC curves. Accordingly, a nomogram was created for intuitive prognosis prediction, which can favorably help clinicians about making the best decisions [19, 21]. The nomogram indicated a more accurate and reliable prognosis prediction of HNSCC by means of mixing SLC20A1 expression and clinical parameters. Consistent with our results, Dong et al. also had identified the SLC20A1 as potential biomarker for the diagnosis and treatment of esophageal adenocarcinoma [22]. Besides, research showed that SLC20A1 overexpression increases pituitary cell proliferation, resulting in an unfavorable prognosis [14]. Jiang et al. found that SLC20A1, among other different genes, was overexpressed, and the progression of tongue squamous cell carcinoma was associated with miR-138 down-regulation [16]. Based on these results, we believe that more research is required to determine the expression level of SLC20A1 in patients with HNSCC, which will be crucial for the translational application of SLC20A1 in HNSCC management.

At present, some studies explored the role of SLC20A1 in different tumor species. In somatotroph adenomas, a positive correlation was noted for tumor recurrence, size, and invasiveness with SLC20A1 levels [23]. Targeted siRNA knockdown of SLC20A1 in breast cancer cells was tested for its influence on tumor sphere formation and cell viability. SLC20A1 was shown to be important in cancer development and to contribute to clinical outcomes in individuals with ER+, claudin-low, and basal-like breast tumors [15]. In esophageal adenocarcinoma, SLC20A1 was considered an independent prognostic indicator for relapse-free survival [22]. To investigate the association of SLC20A1 with HNSCC prognosis, we found that SLC20A1 exaggerated expression promoted the clonal formation and cell proliferation in HNSCC cells. The cell migration and invasion ability after SLC20A1 knockdown are significantly weaker than that of wild cell lines. Therefore, we conclude that high expression of SLC20A1 can remarkably induce tumor cells to invade, migrate, and proliferate, additionally showing the unfavorable prognosis of this biomarker in cases with HNSCC.

Moreover, multidisciplinary treatment for HNSCC is consistent of 3 main approaches: surgery, chemotherapy and radiotherapy [24]. A sensitive chemotherapeutic medicine is important for patients of HNSCC. Widespread use of the platinum-based anticancer drug cisplatin began in 2000, and since then, paclitaxel, among other taxane-based cancer treatments, was develop for HNSCC [25]. Gemcitabine has been shown to be beneficial against HNSCC in many phase II trials, including those combining it with radiation, cisplatin, and docetaxel [26]. Nonetheless, inherent or acquired chemoresistance promotes to adverse prognosis, regional recurrence, and treatment failure. Through the Wnt/-catenin signaling pathway, it has been shown that cisplatin resistance was observed with CD44 high expression levels in HNSCC cells [27]. An increase in the expression of KLF4, ABCG2, and ABCB1 was associated with resistance to paclitaxel in head and neck squamous cell carcinoma (HNSCC), according to research by Duz et al. [28]. Xuan et al. discovered that TGF- levels in cells are a major contributor to resistance to gemcitabine, suggesting that inhibiting TGF- might be an effective new approach for treating HNSCC that has developed resistance to the drug [29]. In our study, we selected three chemotherapeutic medicines including cisplatin, gemcitabine, and paclitaxel for analysis, which were widely used in HNSCC. The findings demonstrated that cases with exaggerated SLC20A1 expression have increased sensitivity to cisplatin and gemcitabine, and reduced sensitivity to paclitaxel, providing insights for further investigation.

Immunotherapy is becoming a breakthrough in oncological therapy for HNSCC [30]. Increasing knowledge about immunological mechanism during carcinogenesis has allowed for the introduction of new therapeutic standards. TIME (tumor immune microenvironment) represents an intricate network. Tumor cells may interact with their inner cytokines, stromal cells, and immune cells to modulate the immunological network, which in turn affects tumor growth and the efficacy of immunotherapy. Immune cell invasion into a tumor has been linked to both immunotherapy response and outcome in head and neck squamous cell carcinoma [31–33]. In this study, the CIBERSORT and TIMER analyses showed that SLC20A1 overexpression was remarkably correlated with TIICs, strengthening what was found about the significant relationship between HNSCC immune microenvironment and SLC20A1. Furthermore, we identified that SLC20A1 overexpression is correlated with more immune exclusion and immune dysfunction and lower TIDE score. Therefore, we suggested that SLC20A1 might take part in the immunotherapy of HNSCC patients. More research is needed to determine SLC20A1's precise role in the tumor-immune milieu, however, since the infiltrative immunological landscape of HNSCC has not been fully described to this point.

To adequately comprehend the participating mechanism of SLC20A1 in HNSCC, we performed GSEA analysis. As was reported, in vascular smooth muscle cells, SM22αgene expression was inhibited and ERK1/2 phosphorylation was promoted due to elevated Pi signaling by SLC20A1 via a Rap1/B-Raf/Mek1/2 cell signaling pathway [34]. Elevated Pi by SLC20A1, which encodes the Pit1 sodium phosphate cotransporter, resulted in phosphorylation of the FGF receptor substrate 2α phosphorylation through the MEK/ERK pathway [35]. The upregulation of SLC20A1 targets the miR-31-5p/MMP3 axis, leading to aggravated degeneration of the extracellular matrix in degenerative human nucleus pulposus cells [36]. In this study, the results indicated that SLC20A1 overexpression had markedly a positive correlation with numerous types of cancer. This data suggests that SLC20A1 has a role in cancer development affects patient outcomes. Furthermore, we observed a robust correlation between high SLC20A1 expression and the P53 signaling route and the MAKP signaling pathway. Important insights into the pathophysiology and therapy of myelosuppression were provided by the discovery of a causal relationship between Pi metabolism and the P53 signaling pathway during myelosuppression. This link was shown to be essential for the survival of hematopoietic stem cells [13]. Research showed the increased sensitivity of SLC20A1-depleted cells to the proapoptotic activity of tumor necrosis factor (the antiapoptotic NFκB pathway is inactivated, while the MAPK pathway is activated) [11].

In addition, we have initiated a preliminary exploration into the mechanisms by which signaling pathways regulate the tumor immune microenvironment. We discovered that SLC20A1 may play a role in modulating the TNF/TNFR2 signaling pathway. TNF, a pleiotropic pro-inflammatory cytokine, is pivotal in numerous cellular events, including cell proliferation, differentiation, and apoptosis [37]. It induces paradoxical effects in the immune system, being critical for both the initiation and coordination of inflammation, while concurrently suppressing immune cell activity. TNF interacts with two distinct receptors, TNFR1 and TNFR2, activating different signaling pathways [38, 39]. TNFR1 primarily promotes inflammatory responses, whereas TNFR2 expression is mainly on activated T cells, especially involving Tregs in immune reactions and stabilizing the CD4+Foxp3+ Treg phenotype, with most CD8+ suppressive T cells also expressing TNFR2. Beyond its role in maintaining Treg proliferation and stability, TNFR2 also acts as an oncogene. TNFR2 expression has been identified in at least 25 different tumor types, including human renal cell carcinoma, multiple myeloma, colorectal cancer, ovarian cancer, and CTCL (cutaneous T-cell lymphoma) [40]. The tumor microenvironment cunningly recruits TNFR2+ Treg cells, which are highly immunosuppressive, thus facilitating tumor immune evasion [41]. Tregs, one of the immunosuppressive cells in the tumor immune microenvironment, control autoimmune responses and inhibit the activation and proliferation of Teffs (effector T cells) through various mechanisms. These include downregulating MHC and co-stimulatory molecules (CD80 and CD86) on APCs (antigen-presenting cells), inhibiting APC maturation, and weakening their interaction with T cells [42]. Tregs can also directly kill T cells and APCs by secreting perforin and granzymes and suppress T cell activation and proliferation by secreting inhibitory cytokines such as TGF-β (transforming growth factor-β), IL (interleukins)-10, IL-35, and depleting γc cytokines [43]. Studies have found that the balance of PD-1 expression between Teffs and Tregs in the tumor immune microenvironment can predict the clinical efficacy of PD-1 inhibitors [44]. Therefore, it is speculated that a decrease in SLC20A1 expression could reduce the activation of the TNF/TNFR2 signaling pathway, thereby inhibiting Tregs, restoring tumor immune response, and eliminating the tumor.

Moreover, we also investigated that high levels of SLC20A1 have a notable association with cell cycle. Because SLC20A1 is a Pi symporter and increases Pi uptake contributing to DNA synthesis and the regulation of the cell cycle [5, 6]. Our study showed that SLC20A1 was significantly related to several metabolism and phosphorylation processes. Hence, it is speculated that SLC20A1 in HNSCC drives the progression of the cell cycle through phosphorylate other proteins.

Although this investigation provides results that might aid to a better comprehension of SLC20A1 expression value in tumor progression and prognosis in HNSCC, different limitations should be highlighted. First, the number of tissue samples could be enlarged. Second, the mechanism and regulation of SLC20A1 expression have not been further explored. Moreover, our study lacks vivo experimental verification.

## CONCLUSIONS

Based on our findings, SLC20A1 is upregulated in HNSCC tissues and may serve as a poor prognostic indication for this disease by encouraging cancer cells to invade, migrate, and proliferate. SLC20A1 overexpression might be closely related with chemotherapy response and is a novel biomarker in the progression of tumors and immune environment in HNSCC, which is worthy of further investigation.

## Supplementary Material

Supplementary Figures
