# Supplementary material for: SLC20A1 is a prospective prognostic and therapy response predictive biomarker in head and neck squamous cell carcinoma
Source: Aging (Albany NY). 2024 Feb 26;16(5):4423–44. doi: 10.18632/aging.205597 (PMC10968711; doi:10.18632/aging.205597)
Supplement: Supplementary Figures [file aging-16-205597-s001.pdf]

SUPPLEMENTARY FIGURES

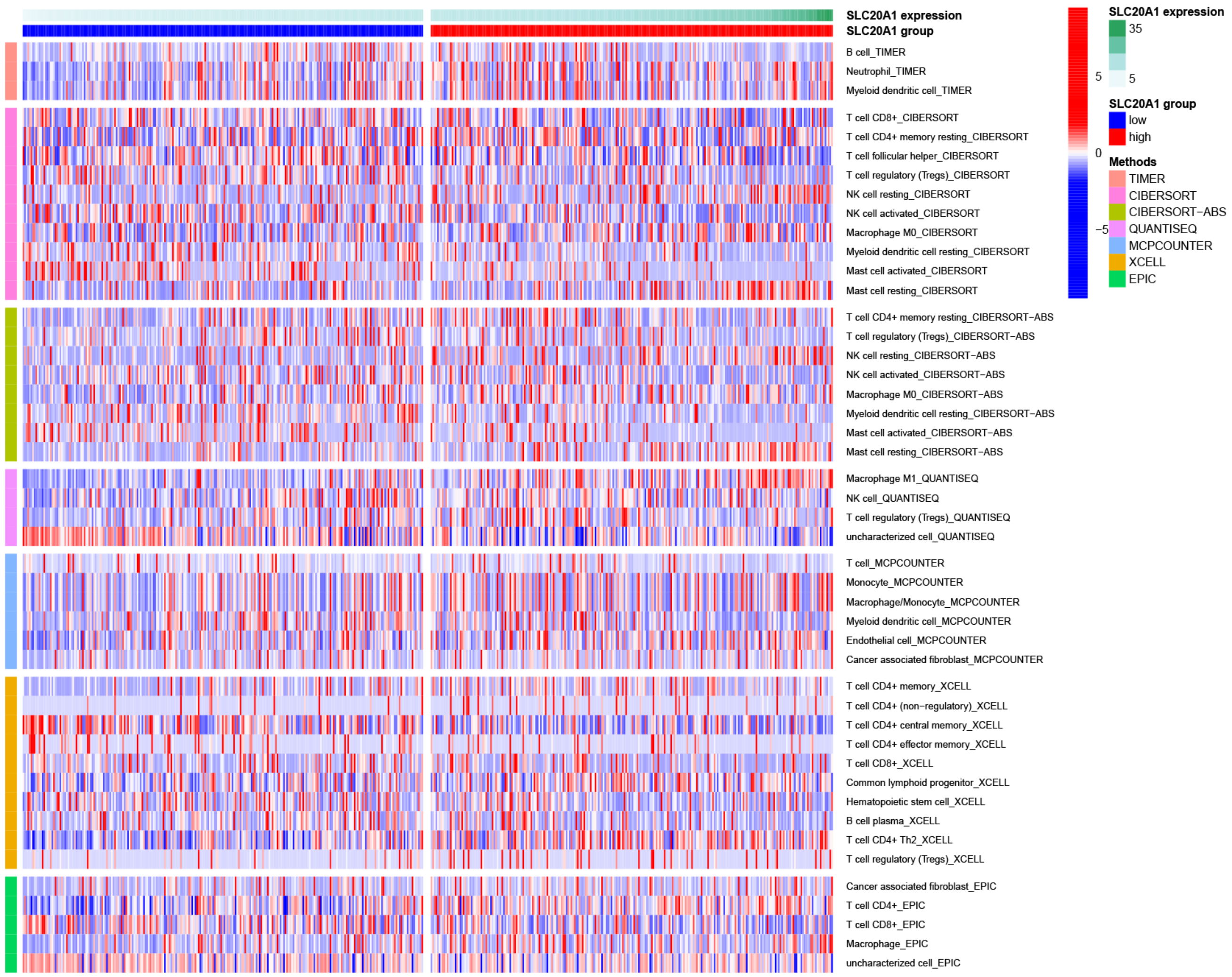

Supplementary Figure 1. Heatmap of SLC20A1 overexpression related to immune cells in HNSCC.

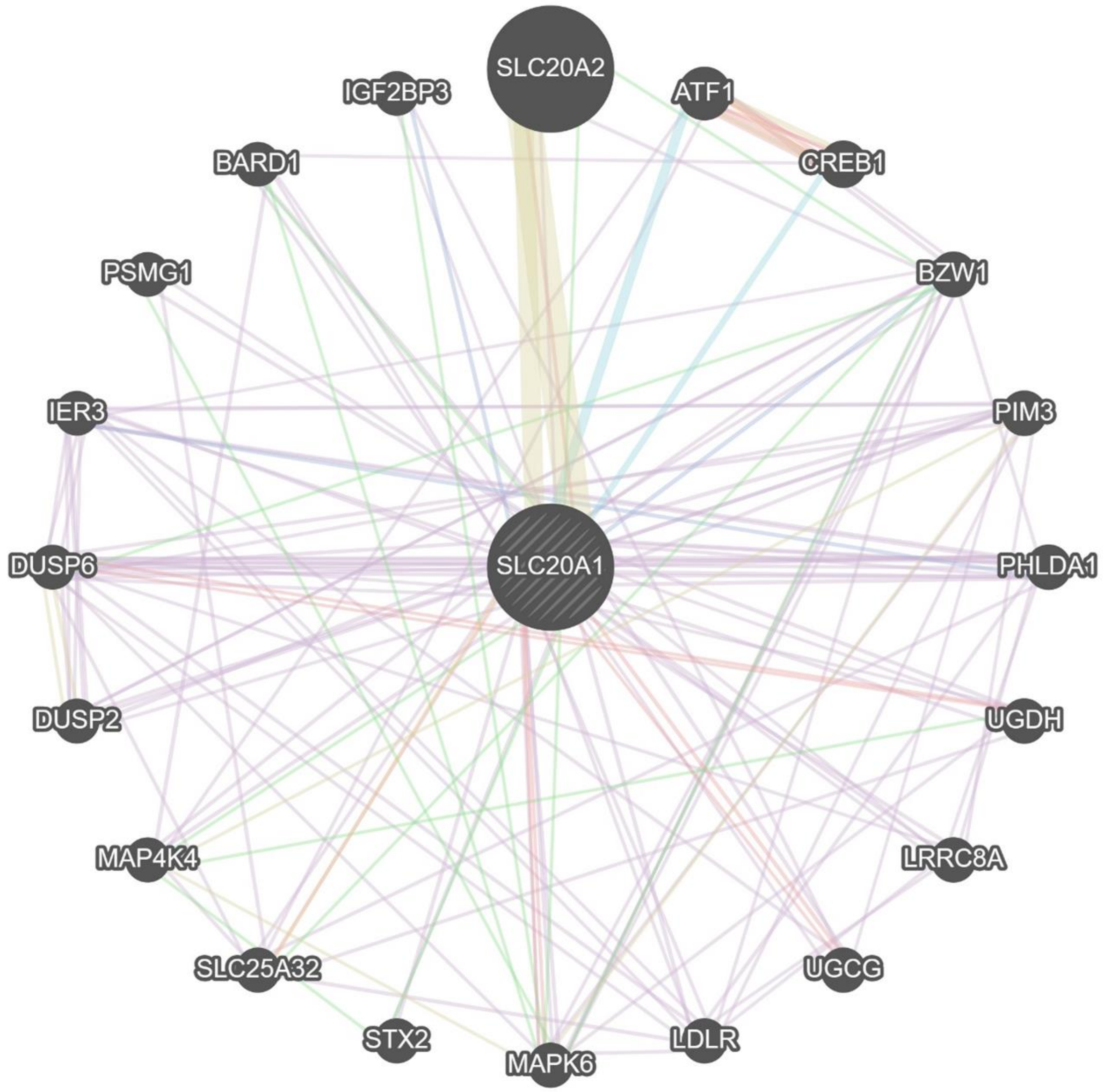

Supplementary Figure 2. Protein-protein interaction network of SLC20A1.

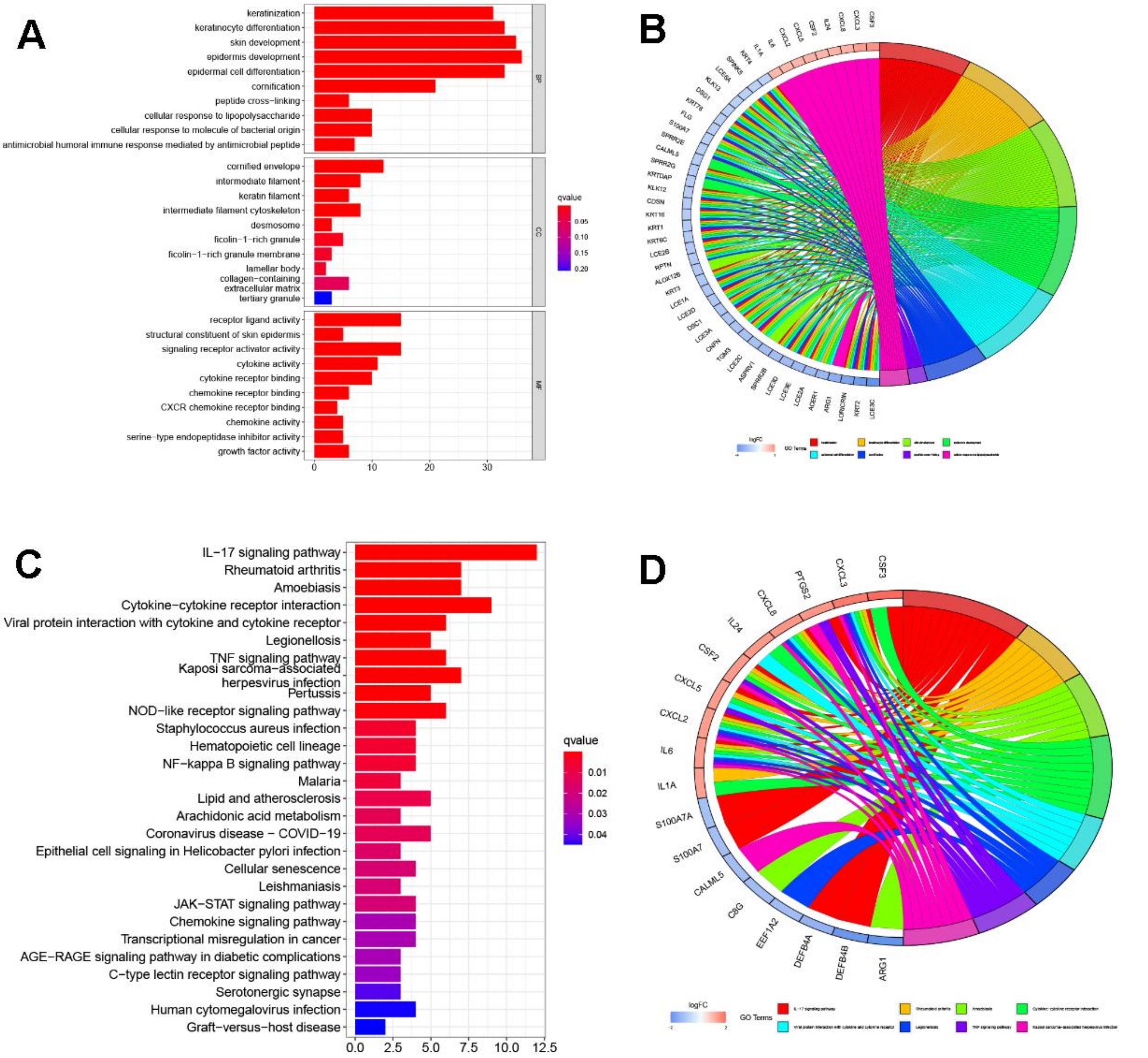

Supplementary Figure 3. Gene function enrichment analysis. (**A**, **B**) Underlying molecular mechanism enriched by SLC20A1 from GO analysis. (**C**, **D**) Biological processes enriched by SLC20A1 from KEGG analysis.

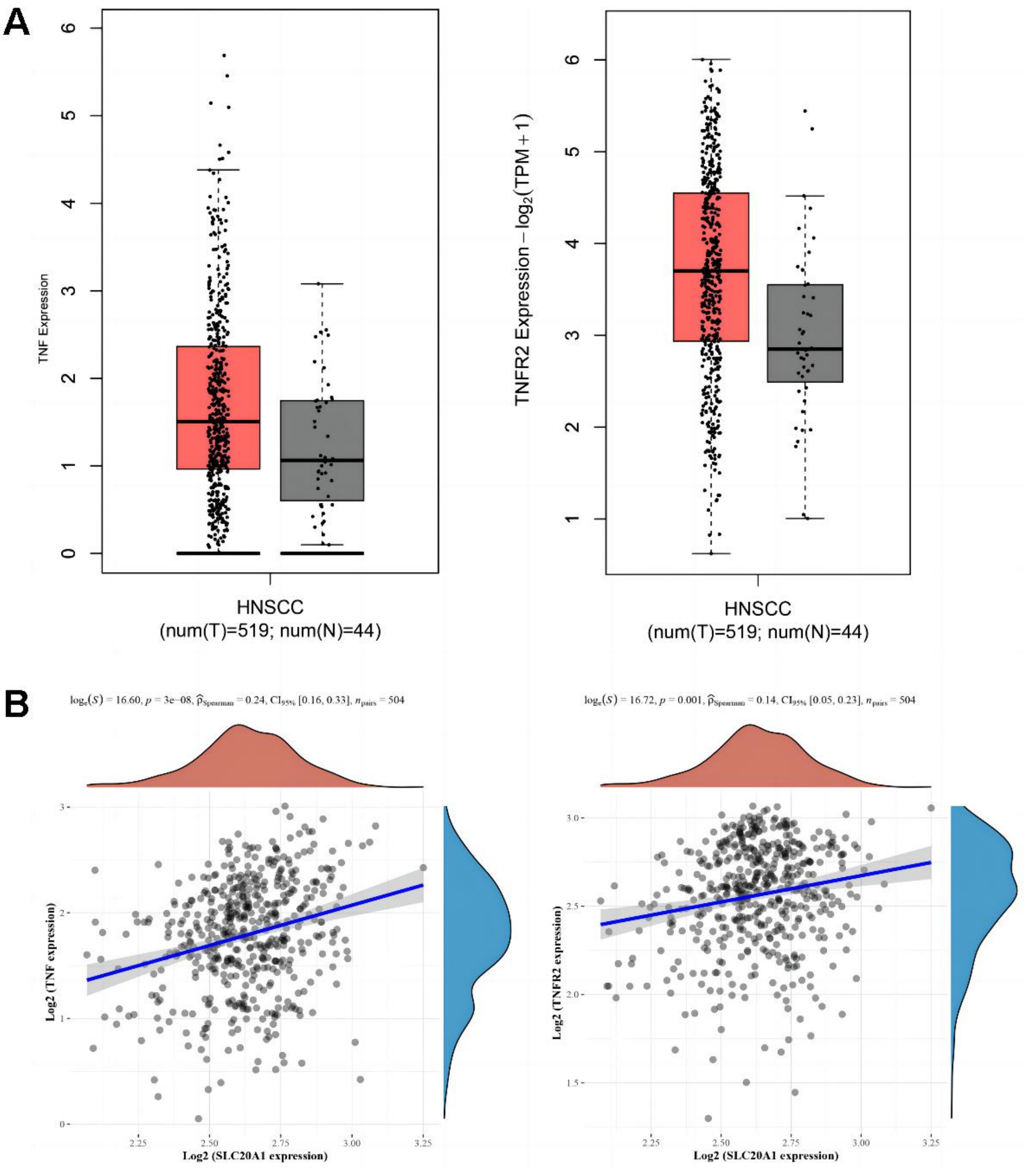

Supplementary Figure 4. Comparative analysis of TNF, TNFR2, and SLC20A1 expression in HNSCC and normal tissues from the TCGA database. (**A**) The expression levels of TNF and TNFR2 in head and neck squamous cell carcinoma and normal tissues; (**B**) The relationship between SLC20A1 expression and the levels of TNF and TNFR2 in head and neck squamous cell carcinoma.

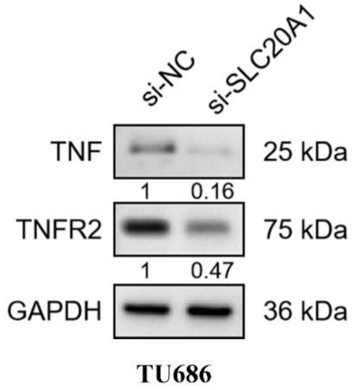

Supplementary Figure 5. Western blot analysis of the relationship between SLC20A1 expression and the levels of TNF and TNFR2 in the head and neck squamous cell carcinoma TU686 cell line.

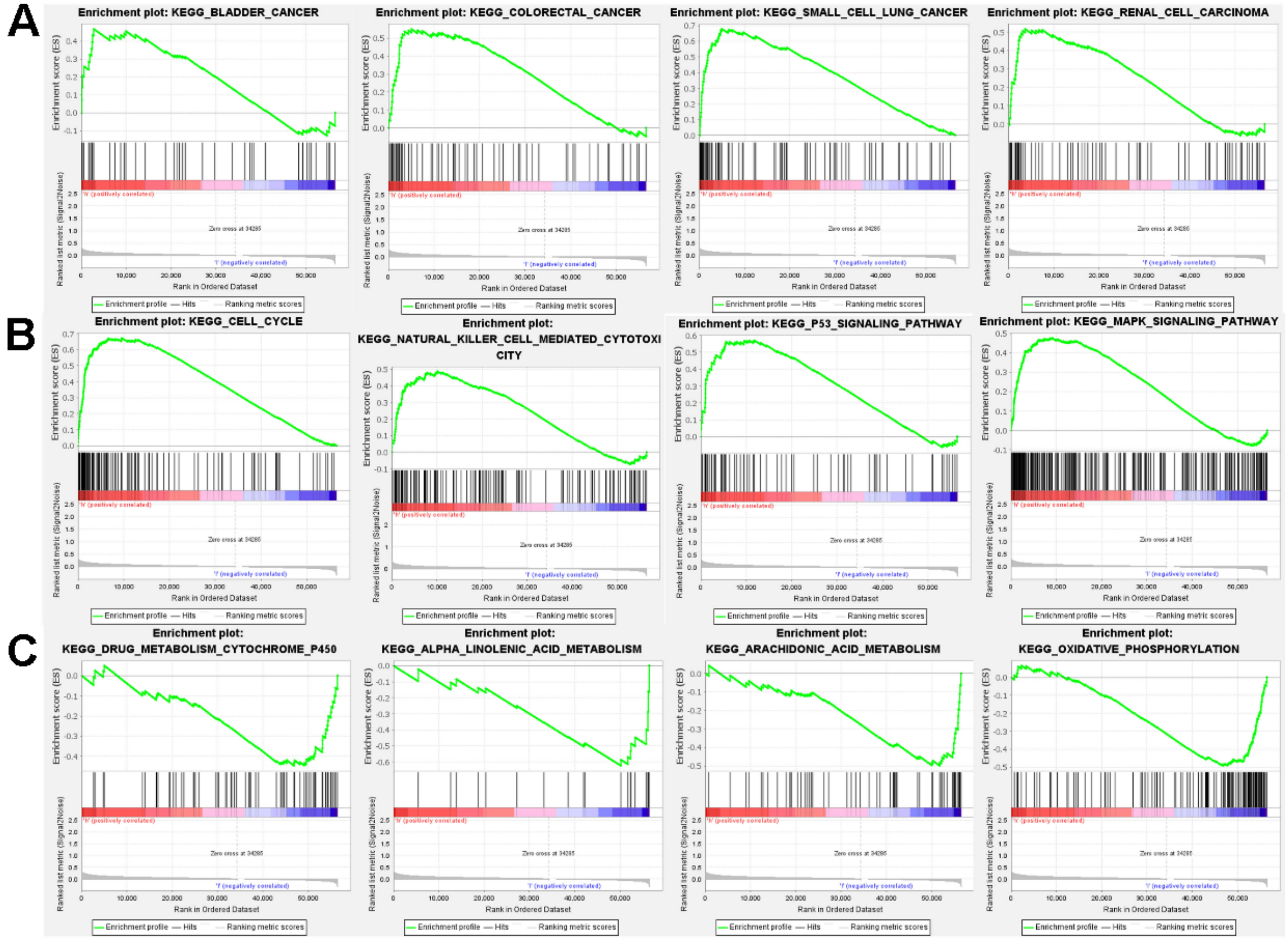

Supplementary Figure 6. SLC20A1-related signaling pathways based on GSEA. (**A**, **B**) GSEA enrichment in the high-SLC20A1 expression phenotype. (**C**) GSEA enrichment in the low-SLC20A1 expression phenotype.
